# A Droplet Digital PCR-Based Approach for Quantitative Analysis of the Adulteration of Atlantic Salmon with Rainbow Trout

**DOI:** 10.3390/foods12234309

**Published:** 2023-11-29

**Authors:** Xiao-Yu Ma, Zhu-Long Shao, Xiao-Ping Yu, Zheng-Liang Wang

**Affiliations:** Zhejiang Provincial Key Laboratory of Biometrology and Inspection and Quarantine, College of Life Sciences, China Jiliang University, Hangzhou 310018, China; xiaoyu20210301@163.com (X.-Y.M.); 17662432876@163.com (Z.-L.S.); yuxiaoping19630306@163.com (X.-P.Y.)

**Keywords:** Atlantic salmon, rainbow trout, adulteration, droplet digital PCR, quantification

## Abstract

Low-cost fish species are often used to adulterate or substitute for Atlantic salmon products, posing a serious threat to market order and public health. Hence, reliable techniques are urgently needed to detect Atlantic salmon adulteration. In this study, a precise method for identifying and quantifying adulterated Atlantic salmon with rainbow trout based on droplet digital PCR (ddPCR) testing was developed. Species-specific primers and probes were designed targeting the single-copy nuclear gene myoglobin of two salmonids. A quantitative formula for calculating the mass fraction of adulterated Atlantic salmon with rainbow trout was established based on a one-step conversion strategy, in which the DNA copy number ratios were directly transformed to meat mass fractions by introducing a fixed constant (the transfer coefficient). The dynamic range of the established ddPCR method was from 1% to 90%, with a limit of detection (LOD) of 0.2% and a limit of quantification (LOQ) of 0.8% for rainbow trout in Atlantic salmon, respectively. The quantification method demonstrated an acceptable level of repeatability and reproducibility, as the values of the relative standard deviation (RSD) for the tested meat mixtures with the known fractions were all less than 5%. Thermal and freezing treatments, as well as adding food additives within the recommended dosage limits, had no significant effect on the quantification accuracy. The method was successfully applied to detect rainbow trout adulteration in commercial raw and processed Atlantic salmon products. In comparison to real-time quantitative PCR (qPCR) testing, the established ddPCR method exhibited a higher level of stability and accuracy. Overall, the ddPCR-based quantitative method exhibited high levels of accuracy, stability, sensitivity, and practicability, suitable for applications in the routine surveillance and quality assurance of salmon products.

## 1. Introduction

Atlantic salmon (*Salmo salar*) is highly appreciated worldwide for its unique taste and rich nutrient content, which consists of significant amounts of polyunsaturated fatty acids and essential trace elements crucial for human health [1,2]. Due to the huge demand for salmon in the Chinese market, foreign imports of Atlantic salmon have rapidly increased in China in recent years. Rainbow trout (*Oncorhynchus mykiss*) is a freshwater fish belonging to the Salmonidae family, which is visually similar to Atlantic salmon in terms of meat color and texture [3]. Due to its rapid growth rate and strong environmental tolerance, rainbow trout is becoming one of the most common cultured fish in China, and hence, its price is much lower than that of Atlantic salmon in the Chinese market. Driven by high economic profits, the substitution of low-cost rainbow trout for high-priced Atlantic salmon as well as its adulteration frequently occur at every stage of the food supply chain [4,5]. These incidents of fraud not only harm consumers’ economic interests, but also increase health and safety risks, as rainbow trout cultured in freshwater may have a high risk of parasite infections [6]. Therefore, efficient and accurate methods for the detection of Atlantic salmon adulterated with rainbow trout are urgently needed.

Traditionally, the identification of rainbow trout and Atlantic salmon mainly relies on their morphological features; however, this is a difficult task since Atlantic salmon is often sold in processed forms in the market, as fillets or blocks, dried or grilled, and canned or smoked. Spectral and chromatographic techniques have been developed and applied to the detection of salmon adulteration, but these suffer from suboptimal levels of specificity and sensitivity [7,8,9]. To date, DNA-based methods are the most widely applied for identifying and quantifying fish adulteration [10,11,12,13,14]. Of these, real-time quantitative polymerase chain reaction (qPCR) testing is the most outstanding representative [15]. Accumulating studies have exploited the qPCR method for the qualitative and quantitative detection of fish adulteration, including the rapid identification of Atlantic salmon, Atlantic cod (*Gadus morhua*), and European plaice (*Pleuronectes platessa*) [16], as well as the accurate quantification of two closely related tuna species (*Thunnus obesus* and *Thunnus albacares*) in a binary mix in tuna cans [17]. The qPCR method can quantify the copy numbers of target DNA by the use of a standard curve based on cycle threshold (Ct) values, which are strongly affected by the amplification efficiency and the purity of the DNA template [18]. Droplet digital PCR (ddPCR) testing is an emerging technique for the precise quantification of nucleic acid, which can provide an absolute measure of DNA content without the need for standard curves, thereby increasing the accuracy of target sequence quantification [19,20]. Nowadays, ddPCR-based methods have been successfully applied for the quantitative detection of various animal derivatives, such as beef, pork, chicken, sheep, horse, turkey, and silver pomfret, in processed and mixed food matrices [21,22,23,24,25,26].

The objective of this study is to develop an efficient and accurate ddPCR method for the identification and quantification of adulterated Atlantic salmon with rainbow trout. To avoid the bias between the measured DNA copy numbers via ddPCR and the actual meat mass fractions, a convenient one-step procedure is adopted to transform the ratio of DNA copies to the weight fraction of meat by introducing a fixed constant that is designated as the transfer coefficient (K) [27]. Consequently, a precise quantitative formula was built and validated for the quantitative detection of the rainbow trout fraction in Atlantic salmon products. An evaluation of the specificity, sensitivity, repeatability, reproducibility, and practicability of the established ddPCR method was also conducted using artificial samples with known proportions and commercially available products. Additionally, qPCR assays were conducted for a quantitative analysis of the adulteration of Atlantic salmon with rainbow trout and the results were compared with those of the ddPCR method. Our results will provide an alternative tool that could be implemented in the routine testing and quality assurance of Atlantic salmon products.

## 2. Materials and Methods

### 2.1. Test Material Preparation

Raw Atlantic salmon and rainbow trout were purchased as whole animals from Zhejiang Beijipin Product Aquatic Products Co., Ltd. and Jingdong Supermarket in Hangzhou, China, respectively. After eviscerating, skinning, and washing them, muscle samples of each fish species were cut into very small pieces using scissors, dried in an oven at 50 °C to a constant weight, and pulverized into powder by a crusher. Different amounts of rainbow trout were added to Atlantic salmon to create binary meat mixtures with known weight percentages in a total mass of 10 g, i.e., 0.01%, 0.05%, 0.1%, 0.2%, 0.5%, 0.8%, 1%, 5%, 10%, 20%, 30%, 40%, 50%, 60%, 70%, 80%, and 90%.

### 2.2. DNA Extraction

Total genomic DNA was extracted from 100 mg of each mixed meat sample using the TIANamp Genomic DNA Kit (Qiagen, Hilden, Germany) according to the manufacturer’s protocol. The purity and concentration of the extracted genomic DNA was measured using a Nanodrop 2000 spectrophotometer (Thermo Fisher Scientific, Waltham, MA, USA). The qualified DNA samples were then diluted to a concentration of 50 ng/μL and stored at −20 °C prior to their use.

### 2.3. Primer and Probe Design

The primers and probes were designed based on the sequences of the single-copy nuclear gene myoglobin (MB) of Atlantic salmon and rainbow trout using software AlleleID^®^ 7.85 (Premier Biosoft, Palo Alto, CA, USA). Probes were 5′-labeled with 5′-hexachlorofluorescein (HEX) or 6-carboxyfluorescein (FAM) as the reporter and 3′-labeled with black hole quencher 1 (BHQ1) as the quencher. Primers and probes were subjected to homologous analysis via BLAST (Basic Local Alignment Search Tool) searches against the GenBank database. Their specificity was further validated by negative control in ddPCR assays. The designed species-specific primers and probes were synthesized using GenScript (Nanjing, China). The primer and probe sequences, target genes, GenBank accession numbers, and amplicon lengths are all provided in Table 1.

### 2.4. ddPCR Procedure

The ddPCR assays were carried out in a total volume of 20 μL. Each reaction mixture contained 10 μL 2 × ddPCR Master Mix (no dUTP) (Bio-Rad, Hercules, CA, USA), 1.8 μL of each primer (10 μM), 0.5 μL of probe (10 μM), 1 μL of template DNA, and 4.9 μL of nuclease-free water (Qiagen, Hilden, Germany). Approximately 20,000 oil droplets were generated using the QX200 droplet generator (Bio-Rad, Hercules, CA, USA) and subsequently transferred to 96-well ddPCR™ plates (Bio-Rad, Hercules, CA, USA). The plates were heat-sealed with a PCR plate heat-seal foil using the PX1^TM^ PCR plate sealer (Bio-Rad, Hercules, CA, USA). PCR testing was performed on a T100™ Thermal Cycler (Bio-Rad, Hercules, CA, USA) using the following cycling parameters: 95 °C for 10 min, followed by 39 cycles of 94 °C for 30 s, 58 °C for 60 s, and finally 98 °C for 10 min. After PCR amplification, the QX200 droplet reader was used to measure the fluorescence signal of each droplet, and the absolute copy numbers per reaction were calculated with software QuantaSoftV 1.7.4.0971 according to Poisson distribution [28].

### 2.5. Specificity

To evaluate the specificity of the ddPCR system, DNA samples from two target species (Atlantic salmon and rainbow trout) and eight non-target species were extracted using the method mentioned above and then used as template for ddPCR assays. The non-target species include Atlantic cod (*Gadus morhua*), Sockeye salmon (*Oncorhynchus nerka*), Chinook salmon (*Oncorhynchus tshawytscha*), Coho salmon (*Oncorhynchus kisutch*), Chum salmon (*Oncorhynchus keta*), northern whitefish (*Coregonus peled*), Arctic char (*Salvelinus alpinus*), and brown trout (*Salmo trutta*).

### 2.6. Establishment of Quantitative Formula

The ddPCR system measures the absolute copy numbers of target DNA sequence in tested meat samples; however, these cannot represent the meat mass fractions directly since the cell density and genome size are different among fish species. To avoid this deviation, transfer coefficient, a fixed constant designated as K, was introduced to directly convert the DNA copy number ratio to the meat mass fraction as described previously [27]. In brief, the mass ratio between rainbow trout and Atlantic salmon in the binary meat mixtures can be calculated by the following formula:

$$\frac{M_{O}}{M_{S}}=\frac{\frac{Q_{O}}{C_{O}}}{\frac{Q_{S}}{C_{S}}}=\frac{C_{S}}{C_{O}}\times \frac{Q_{O}}{Q_{S}},$$

where M_O_ and M_S_ refer to the mass of rainbow trout and Atlantic salmon, respectively; Q_O_ and Q_S_ represent the *MB* gene copy number of rainbow trout and Atlantic salmon estimated via ddPCR measurements, respectively; and C_O_ and C_S_ indicate the copy number of *MB* gene per unit mass of rainbow trout and Atlantic salmon, respectively. For a given fish species, the copy number of a single-copy gene per unit mass can be considered invariable. Therefore, C_S_/C_O_ would be a constant under fixed experimental conditions and was designated as transfer coefficient (K). Consequently, the above formula for quantifying the adulteration of rainbow trout in Atlantic salmon products could be represented as follows:

$$\frac{M_{O}}{M_{S}}=K\times \frac{Q_{O}}{Q_{S}}.$$

To determine K values, binary meat mixtures containing rainbow trout with the mass fractions of 10%, 30%, 50%, 70%, and 90% were used for ddPCR quantification of the species-specific *MB* gene. Each sample was tested in six replicates.

To verify accuracy and stability of derived K values, ddPCR assays were performed using binary mixtures with known mass fractions of rainbow trout of 20%, 40%, 60% and 80%, respectively. Each sample was tested in quadruplicate.

### 2.7. Dynamic Range, Limit of Detection (LOD), and Limit of Quantification (LOQ)

To evaluate the dynamic range of quantification, a series of mixed samples containing rainbow trout with mass fractions ranging from 1% to 90% were used in multiple ddPCR assays. By plotting the actual mass fractions (X-axis) and the ddPCR-measured values of the mass ratios (Y-axis), linear regression line was established and the regression coefficient (R^2^) was estimated. To determine the LOD and LOQ, ddPCR assays were performed using mixed samples containing rainbow trout with the lower mass fractions of 0.05%, 0.1%, 0.2%, 0.5%, 0.8%, 1%, and 5%, respectively. Each sample was tested in quadruplicate.

### 2.8. Repeatability and Reproducibility

To determine the repeatability of the established ddPCR method, six replicates of ddPCR assays were conducted for each mixed sample containing rainbow trout with the mass fractions of 10%, 30%, 50%, 60%, and 80%, respectively, by two operators in our lab. The reproducibility of the ddPCR method was determined using the same samples as above. Independent experiments were performed on three consecutive days by two operators.

### 2.9. Evaluation of the Impact of Treatment Temperatures

To determine the impact of thermal and freezing treatments on the robustness of the ddPCR assays, binary mixed samples containing rainbow trout with the mass fractions of 10% and 70% were steamed in a digital thermostatic water bath at 100 °C for 5, 10, and 20 min or frozen in a refrigerator at −20 °C for 12, 24, and 72 h, respectively. Each sample was tested in quadruplicate.

### 2.10. Evaluation of the Impact of Different Food Additives

To determine the impact of different additives on the robustness of the ddPCR assays, four kinds of food additives (sodium glutamate, β-carotene, carrageenan, and fish powder flavor) were separately mixed with binary meat mixtures containing 70% rainbow trout. The final mass fraction was set to 0.2% for sodium glutamate (recommended dosage: 0.1–0.3%), 0.4% for β-carotene (recommended dosage: 0.3–0.5%), 0.5% for carrageenan (recommended dosage: 0.3–0.8%), and 0.5% for fish powder flavor (recommended dosage: 0.1–1%). Each sample was tested in quadruplicate.

### 2.11. Testing of Commercially Available Products

Sixteen commercially processed products labeled as Atlantic salmon purchased in local supermarkets, shops, and various sales platforms were used to test the practicability of the established ddPCR-based quantification method. A wide range of product types were selected, including raw, dried, frozen, canned, smoked, toasted, etc. For each sample, three replicates were performed. The ddPCR-estimated contents of Atlantic salmon were then compared with those declared on the product labels.

### 2.12. qPCR Assays

For the method validation and the comparison of quantitative results, qPCR assays were performed using a CFX384 Touch Real-Time PCR Detection System (Bio-Rad, Hercules, CA, USA) with Premix Ex Taq^TM^ (Probe qPCR) (Takara, Japan). Each reaction mixture with a total volume of 20 μL contained 10 μL 2 × Premix Ex Taq^TM^ (Probe qPCR), 0.4 μL of 50 × ROX reference dye, 0.4 μL of each primer (10 μM), 0.8 μL of probe (10 μM), 2 μL of template DNA, and 6 μL of nuclease-free water (Qiagen, Hilden, Germany). Amplification conditions were as follows: 95 °C for 20 s, followed by 40 cycles at 95 °C for 5 s and 58 °C for 30 s. Two standard curves for rainbow trout and Atlantic salmon were constructed using six four-fold serially diluted DNA (50–0.05 ng/μL) extracted from pure meat samples, respectively. Binary mixtures with known mass fractions of rainbow trout (20%, 40%, 60%, and 80%) and sixteen commercial products were selected for qPCR assays. The Ct values determined from qPCR system were interpolated on the corresponding standard curve to yield the DNA concentrations of species-specific gene (*MB*). The relative quantity of rainbow trout (P_O_) in the tested samples was calculated as follows: P_O_ (%) = C_O_/(C_O_ + C_S_) × 100, where C_O_ and C_S_ refer to the concentrations of rainbow trout and Atlantic salmon DNA, respectively. Each sample was tested in quadruplicate.

### 2.13. Statistical Analysis

DPS software v7.05 was used for statistical analysis [29]. All data from the repeated experiments are expressed as mean ± standard deviation. A one-way analysis of variance (ANOVA) was performed to evaluate the impacts of thermal and freezing treatments, as well as the addition of food additives on the ddPCR quantification results. Differences were considered to be significant at *p* < 0.05. Linear regression analysis was performed using GraphPad Prism 8.

## 3. Results and Discussion

### 3.1. Specificity

Mitochondrial DNA (mtDNA) is a well-established molecular target for qualitative species identification during the process of the detection of food adulteration [21,22,23]. As to whether mtDNA can be used for the quantitative detection of adulteration is disputed. Since the amount of mitochondria per cell varies with animal species and tissue types, mtDNA-based detection would inevitably lead to either an under- or over-estimated result. For instance, fresh pig liver was found to result in three times more target DNA copies per unit mass in procine ddPCR assays when compared to those of fresh muscle samples [22]. An effective alternative is to use single-copy genes as detection targets, since their expression levels are relatively stable among different tissues and individuals [30,31,32]. In the present study, the single-copy nuclear gene myoglobin (MB) of Atlantic salmon and rainbow trout was chosen as the target gene for the quantitative detection.

**Figure 1:**
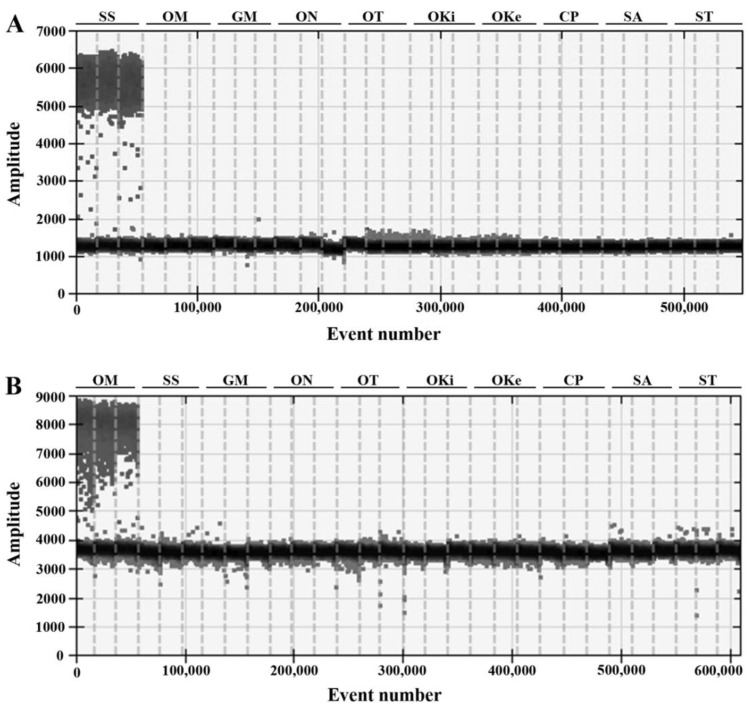
The specificity of primers and probes for Atlantic salmon (**A**) and rainbow trout (**B**) in the ddPCR assays. The horizontal axis represents the number of droplets measured and the vertical axis indicates the amplitude measured per droplet. The non-target fish species were all with no amplification. SS: *Salmo salar*, OM: *Oncorhynchus mykiss*, GM: *Gadus morhua*, ON: *Oncorhynchus nerka*, OT: *Oncorhynchus tshawytscha*, OKi: *Oncorhynchus kisutch*, OKe: *Oncorhynchus keta*, CP: *Coregonus peled*, SA: *Salvelinus alpinus*, and ST: *Salmo trutta*.

To confirm the specificity of the primers and probes, ten species including Atlantic salmon, rainbow trout, and eight other negative control fish species were tested. As shown in Figure 1, positive signals were only detected in the ddPCR assays using the DNA template of the corresponding target species, while cross-reactions with any non-target species were not determined. These results indicate that the sets of the primers and probes were species-specific and are suitable to be used for identifying and quantifying rainbow trout derivatives in Atlantic salmon products.

### 3.2. Establishment of Quantitative Formula

As mentioned above, the difficulty in the quantification of meat adulteration is converting the DNA copy number ratios to the mass fractions. Nowadays, most of studies adopted a two-step conversion method to quantify meat adulteration by first establishing a standard curve of the DNA content versus the DNA copy number and then building a standard curve of the meat mass versus the DNA content [26,33,34]. Obviously, this method would increase the experimental complexity and the deviation of the quantification results. In this study, we developed a ddPCR system to quantify the mass fraction of target species with a one-step conversion procedure by using a transfer coefficient (K).

To estimate the K value, binary meat mixtures with five mass fractions (10%, 30%, 50%, 70%, and 90%) were used for the ddPCR quantification of the species-specific MB gene. The results showed that the average K value for the five different mass fractions was 0.43 with a relative standard deviation (RSD) of 3.02%, indicating that the K value was stable against any mass fraction of rainbow trout versus Atlantic salmon (Table 2). Furthermore, mixed samples with known mass fractions (20%, 40%, 60%, and 80%) were subjected to ddPCR testing for the verification of the accuracy and stability of the K value. As shown in Table 3, the measured results based on the estimated K value were consistent with the actual mass ratios. The absolute value of the deviation ranged from 0.60% to 3.96% with an average of 2.53%, highly suggesting that the estimated K value was quite stable and accurate. Thus, a precise quantitative formula was finally established for the quantification of adulterated Atlantic salmon with rainbow trout, which was represented as M_O_/M_S_ = 0.43 × (Q_O_/Q_S_).

### 3.3. Dynamic Range, LOD, and LOQ

**Figure 2:**
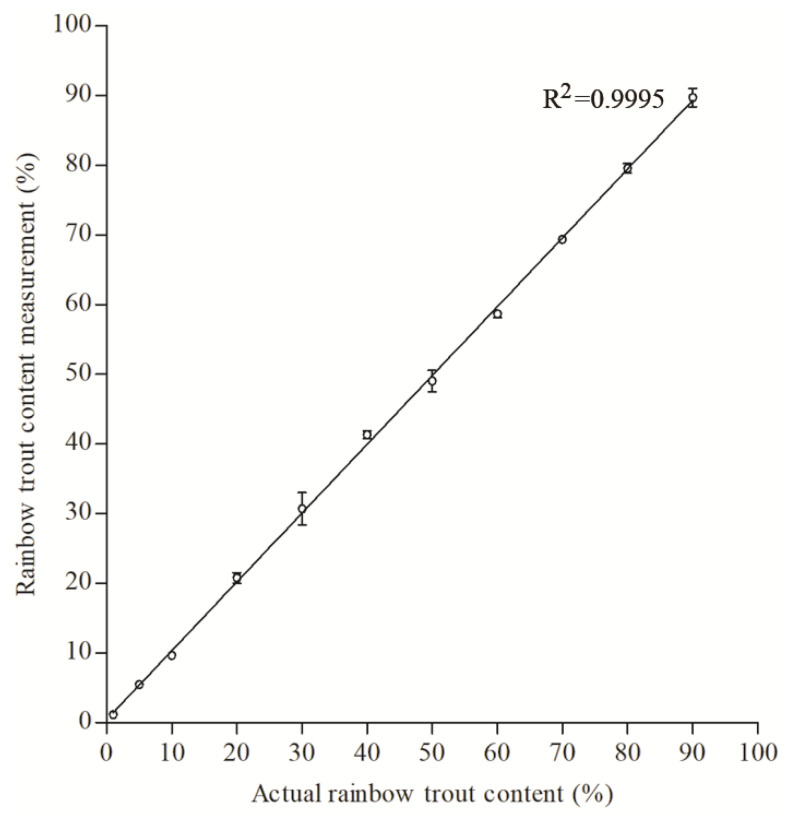
The linearity range of the ddPCR assays for quantification of rainbow trout fraction in Atlantic salmon. The horizontal axis represents the actual mass proportion of rainbow trout in Atlantic salmon and the vertical axis indicates the measured mass proportion of rainbow trout in Atlantic salmon via the ddPCR assays. The mass proportion of rainbow trout in Atlantic salmon is distributed over the range of 1%, 5%, 10%, 20%, 30%, 40%, 50%, 60%, 70%, 80%, and 90%. Error bars indicate standard deviation of the mean from four replicates. The correlation coefficient (R^2^) of the linear regression between the actual and measured content was 0.9995.

The dynamic range of the established ddPCR system was determined using a series of mixed meat samples containing rainbow trout with mass fractions varying in the range of 1% to 90%. As shown in Figure 2, the content of rainbow trout was showed a good recovery from the lowest (1%) to the highest level (90%). The absolute values of the deviation between the measured values via ddPCR and the actual contents were all below 5%, except for mixed samples with a lower content of rainbow trout. The deviation value was ~15% for binary mixtures with 1% rainbow trout and ~10% for mixed samples containing 5% rainbow trout, respectively. The correlation coefficient (R^2^) of the linear regression between the actual content and measured content was 0.9995 (*p* < 0.0001), indicating that the established ddPCR system had a wide dynamic range ranging from 1% to 90%. These results were similar to the findings observed in a previous study on the ddPCR quantification of the adulteration of the meat of fur-bearing animals, in which the dynamic range was between 1% and 90% for fox, mink, and raccoon [35]. Noticeably, the deviation values detected in this study were much lower than those determined in previous studies, which used a two-step conversion method based on ddPCR for quantifying meat adulteration [26,33]. For instance, the deviation values for 5–90% pork in beef/pork binary mixtures varied from −21.88% to 23.33%, while the average deviation detected in the present study was as low as 2.90% [33]. These results indicate that the ddPCR method adopting a one-step conversion strategy could exhibit a relatively higher accuracy than that of the method using a two-step conversion procedure.

The LOD in the present study is defined as the lowest mass fraction of rainbow trout in Atlantic salmon that could be stably detected. As shown in Table 4, positive signals could be stably observed in the mixed samples containing rainbow trout with a mass fraction of 0.2%. Hence, the LOD of the established ddPCR method was set to 0.2% for rainbow trout in Atlantic salmon. The LOQ is the lowest mass fraction of rainbow trout in Atlantic salmon for which the ddPCR method provides results with an acceptable level of uncertainty. According to the Codex Alimentarius Guidelines CAC/GL 74-2010 [36], the level of acceptable uncertainty was chosen at a maximum deviation of 25%. Based on this criterion, the LOD of the established ddPCR method was determined to be 0.8%, which was parallel to the results of recently published research on the ddPCR-based quantitative detection of meat adulteration adopting a one-step conversion strategy to transform the ratios of DNA copy numbers into mass fractions of meat [31,35]. Overall, the ddPCR method established in the present study for quantifying the adulteration of Atlantic salmon with rainbow trout was highly sensitive and accurate.

### 3.4. Repeatability and Reproducibility

**Figure 3:**
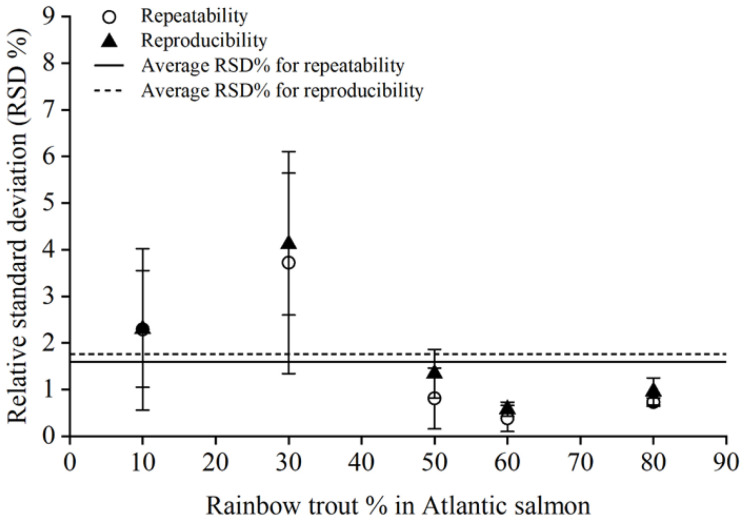
Repeatability and reproducibility of the ddPCR assays for rainbow trout fraction in Atlantic salmon. Relative standard deviations (RSD%) are presented for measured values from five mixed samples containing rainbow trout with the mass proportions of 10%, 30%, 50%, 60%, and 80% in ddPCR assays. The solid line and dashed line represent average RSD for repeatability among ddPCR replicates and for reproducibility among independent experiments, respectively.

To determine the repeatability and reproducibility of the method, a ddPCR analysis was performed on six sets of mixed samples with different fractions (10%, 30%, 50%, 70%, and 80%) by two different experienced operators under the same experimental conditions for three consecutive days. As illustrated in Figure 3, the RSDs that were determined for repeatability among the ddPCR replicates and for reproducibility among the independent experiments were all less than 5%, which were much lower than the acceptance criterion of ≤25%. The highest values of RSD for repeatability and reproducibility were observed for the mixed samples containing rainbow trout with a mass fraction of 30%. The average RSD was 1.59% for repeatability and 1.76% for reproducibility, respectively. These results demonstrated that the established ddPCR method exhibited a high level of repeatability and reproducibility.

### 3.5. Impact of Treatment Temperatures on Quantification

**Figure 4:**
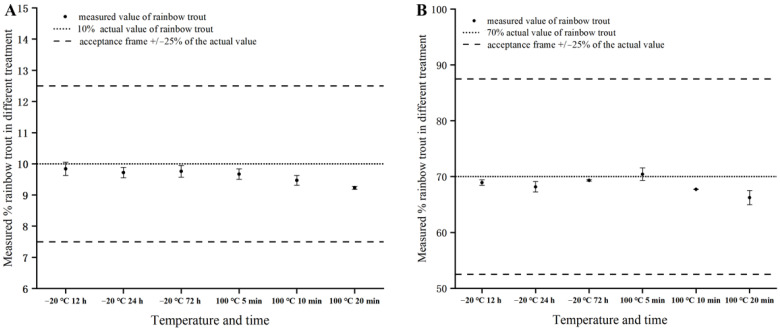
Effect of temperature on the accuracy of ddPCR quantification of 10% (**A**) and 70% (**B**) rainbow trout in Atlantic salmon. The dotted line indicates the actual mass proportion of rainbow trout in Atlantic salmon. The dashed lines represent the acceptability criterion of a maximum deviation of 25%. Error bars indicate standard deviation of the mean from four replicates.

Thermal treatments, such as baking, cooking, roasting, or frying, are commonly used in the meat processing industry to improve the taste and safety of meat products, while freezing extends the shelf life of raw and processed fish products and is widely used for their storage and transportation [37,38,39]. As is well known, the environmental temperature, especially for high temperatures, is an important factor affecting the stability of genomic DNA [40]. To assess the impact of thermal and freezing treatments on the robustness of the quantification method, different temperature treatments, including 100 °C for 5, 10, and 20 min and −20 °C for 12, 24, and 72 h, for mixed meat samples with two known mass fractions (10% and 70%) were implemented. The absolute values of deviation in the measurement of the binary mixtures containing 10% and 70% rainbow trout that were treated under −20 °C varied from 0.96% to 2.79%, with an average of 1.97%. The freezing treatment led to an underestimation of the results both for the mixed samples with 10% and 70% rainbow trout, in agreement with the results detected in a recent study focusing on Atlantic salmon quantification [30]. Obviously, the stability of genomic DNA was negatively impacted in the meat samples under freeze/thaw actions during DNA extraction, leading to a decrease in the accuracy of the quantification results. Compared to the low-temperature-treated group, the deviation values were observed to be slightly higher than those in the samples treated with a high temperature. The highest value of deviation in the measurement of mixed meat samples under the thermal treatment was −7.70% and the mean absolute deviation was 4.24%, indicating that the thermal treatment exerted relatively higher negative impacts on the accuracy of the ddPCR assays. Nevertheless, no significant impact was observed on the measured values from the ddPCR assays under the thermal and freezing treatments (*p* > 0.05). The deviation values for all tested samples were well within the acceptance criterion of ≤25% (Figure 4). It is thus clear that the established ddPCR method maintained a high level of stability and accuracy. Similar results were also found in recent studies focusing on the ddPCR-based quantification of meat adulteration [27,31].

### 3.6. Impact of Food Additives on Quantification

Food additives are trace substances that are intentionally added to food to maintain or improve its taste, texture, flavor, and appearance, as well as extend its shelf life [41,42]. Processed seafood for sale, including Atlantic salmon products, invariably contains food additives. To assess the impact of food additives on the robustness of the quantification method, four different additives within the recommended dosage limits were artificially mixed into binary meat mixtures of rainbow trout and Atlantic salmon and subjected to ddPCR assays. As shown in Table 5, the RSDs and deviation in the measurement of the 70% rainbow trout mixtures with four additives were all below 3%. The absolute values of the deviation between the measured values via ddPCR and the actual contents were 2.52% for sodium glutamate, 1.92% for β-carotene, 2.99% for carrageenan, and 1.37% for fish powder flavor, respectively. The ANOVA results revealed that the tested additives exerted no significant impact on the stability and accuracy of the established ddPCR method (*p* > 0.05), which is consistent with the results reported by Yu et al. [35].

### 3.7. Comparsion of the ddPCR and qPCR Assays

**Figure 5:**
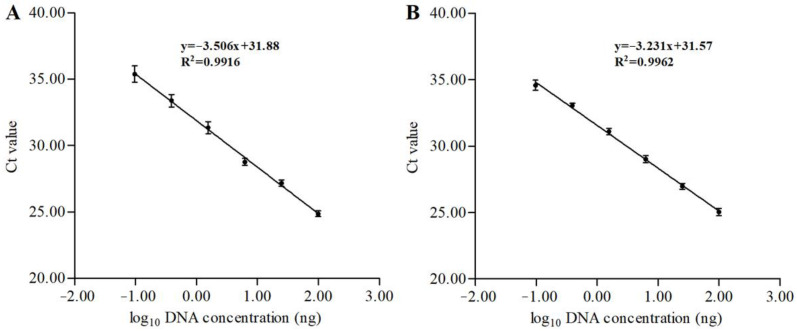
Standard curves for the species-specific gene (myoglobin-encoding gene *MB*) of rainbow trout (**A**) and Atlantic salmon (**B**) by plotting log_10_ of the DNA concentrations (the horizontal axis) and Ct values (the vertical axis) in the qPCR assays. DNA extracted from pure meat sample was serially diluted to obtain final concentrations ranging from 50 to 0.05 ng/μL. Error bars indicate standard deviation of the mean from four replicates. The correlation coefficient (R^2^) of the linear regression was 0.9916 for rainbow trout and 0.9962 for Atlantic salmon, respectively.

To evaluate the accuracy and stability of the established ddPCR method, binary meat mixtures containing rainbow trout with the mass fractions of 20%, 40%, 60%, and 80% were also subjected to qPCR assays, a method which has been widely applied in the detection of meat adulteration [15,16,17]. The standard curves for rainbow trout and Atlantic salmon were independently established by plotting the log_10_ values of the DNA concentrations (X-axis) and Ct values (Y-axis). Accordingly, the following two equations were defined: y_1_ = −3.506x_1_ + 31.88 for rainbow trout and y_2_ = −3.231x_2_ + 31.57 for Atlantic salmon, where y_1_ and y_2_ represented the Ct values determined in the specific qPCR system for the rainbow trout and Atlantic salmon, respectively; and x_1_ and x_2_ represented the log_10_ of the DNA concentrations of the rainbow trout and Atlantic salmon, respectively (Figure 5). The linear correlation (R^2^) and amplification efficiency were 0.9916 and 92.85%, respectively, for the rainbow trout, and 0.9962 and 103.94%, respectively, for the Atlantic salmon. Consequently, the relative quantity of rainbow trout in the tested samples was estimated and compared with that measured via the ddPCR method. As shown in Table 3, the RSD values determined in both the ddPCR and qPCR assays were all less than 10%. However, the RSD values detected in the qPCR assays were much higher than those determined in the ddPCR assays (*p* < 0.05). The absolute values of deviation from the qPCR assays were also significantly higher than those from the ddPCR assays (*p* < 0.05), which is consistent with the results observed in a previous report [27]. The deviation values in the measurement of mixed meat samples with known mass fractions (20%, 40%, 60%, and 80%) ranged from −8.02% to 12.77% in the qPCR assays. The mean absolute deviation was 10.61%, which was much higher than that estimated by the ddPCR method (2.53%), indicating that the ddPCR method exhibited higher levels of accuracy and stability than the qPCR approach in the quantitative detection of the adulteration of Atlantic salmon with rainbow trout.

### 3.8. Quantitative Testing of Commercially Available Products

To verify the application of the established quantitative method, sixteen retail Atlantic salmon products were tested and the detection results are summarized in Table 6. Of those, three samples were adulterated with rainbow trout. Two of them (salmon fish balls and salmon fish floss) contained rainbow trout with mass fractions of 62.50% and 89.14%, which were calculated based on the established quantitative formula, respectively. For example, no Atlantic salmon content was detected in a raw meat product labeled as fresh salmon sashimi, even though the manufacturer declared the content was 100% Atlantic salmon. Similar results were also determined using the qPCR assays. These results confirmed that the developed ddPCR method displayed a good level of practicability.

## 4. Conclusions

In the present study, an accurate and reliable ddPCR-based quantification method was developed for the detection of the adulteration of Atlantic salmon with rainbow trout products. Species-specific primers and probes were designed for rainbow trout and Atlantic salmon targeting the single-copy nuclear gene myoglobin. The quantitative formula M_O_/M_S_ = 0.43 × (Q_O_/Q_S_) was established to quantify the mass fraction of adulterated Atlantic salmon with rainbow trout by a one-step transformation of the gene copy number ratios by introducing a transfer coefficient. Our linear regression established a detection range between 1% and 90% for the rainbow trout/Atlantic salmon mixtures. The LOD and LOQ for the quantification method were 0.2% and 0.8%, respectively. The method exhibited high levels of stability and accuracy under thermal and freezing treatments, as well as the addition of food additives. The developed method also displayed a good level of practicability for routine analyses of commercially available Atlantic salmon products. Compared to the qPCR approach, the developed ddPCR method achieved higher levels of accuracy and stability. Overall, the ddPCR method established in the present study can be a valuable tool for market regulators in the routine detection of Atlantic salmon adulteration.

## Figures and Tables

**Table 1:** Specific primers and probes used for the ddPCR assay.

Species	Target Gene	GenBank Accession Number	Primer/Probe	Sequences (5′-3′)	Amplicon Length
Atlantic salmon	myoglobin	NM_001140642	SS-F	GAGAGGTCACAGGGATAGGA	93 bp
			SS-R	CAAACCAGCACTTAGAATTTAC	
			SS-P	HEX-AACTGGAAACTTACATTTGAAGCAG-BHQ1	
Rainbow trout	myoglobin	NM_001171862	OM-F	TTGCTTGTGACTTCCAGA	141 bp
			OM-R	AGAGGAACAACGCACATT	
			OM-P	FAM-ACTGGAAAAGTGTATGAGGCAAAGC-BHQ1	

**Table 2:** The ratio of DNA copy numbers of unit mass with different fractions of rainbow trout, respectively.

Mass Proportion, %	Rainbow Trout Test (*n* = 6), Copies/μL	Atlantic Salmon Test (*n* = 6), Copies/μL	K Value	K Mean	RSD ^a^, %
10	94.00 ± 0.89	376.5 ± 2.88	0.44	0.43	3.02
30	101.23 ± 4.87	97.79 ± 1.68	0.41		
50	121.30 ± 2.87	51.57 ± 1.51	0.43		
70	183.50 ± 1.87	34.78 ± 0.86	0.44		
90	226.50 ± 2.74	11.13 ± 0.77	0.44		

^a^ The relative standard deviation of the calculated K values.

**Table 3:** Quantification results of binary meat mixtures containing rainbow trout with known mass proportions based on the calculated K value.

Actual Value, %	Measured Value, %		RSD ^a^, %		Deviation ^b^, %	
	ddPCR	qPCR	ddPCR	qPCR	ddPCR	qPCR
20	20.79 ± 0.48	17.76 ± 1.75	2.29	9.85	3.96	−11.18
40	41.32 ± 0.36	45.11 ± 3.10	0.87	6.88	3.30	12.77
60	58.65 ± 0.33	53.73 ± 2.73	0.56	5.08	−2.25	−10.46
80	79.52 ± 0.44	73.58 ± 3.28	0.55	4.46	−0.60	−8.02

^a^ The relative standard deviation of the content of rainbow trout measured via ddPCR and qPCR, respectively. ^b^ The deviation of the mean content of rainbow trout measured via ddPCR and qPCR compared to the actual value, respectively.

**Table 4:** Limit of detection (LOD) and limit of quantification (LOQ) of the ddPCR assay for rainbow trout fraction in Atlantic salmon.

Actual Value, %	Measured Value ^a^, %	RSD ^b^, %	Detection Rate ^c^, %	Deviation ^d^, %
0.05	0	-	0	100
0.1	0.15 ± 0.12	81.28	75.00	51.71
0.2	0.36 ± 0.08	22.78	100	81.89
0.5	0.37 ± 0.03	8.85	100	−25.52
0.8	0.76 ± 0.06	6.36	100	−4.52
1	1.15 ± 0.05	4.46	100	15.28
5	5.51 ± 0.31	5.71	100	10.10

^a^ The content of rainbow trout measured via ddPCR. ^b^ The relative standard deviation of the content of rainbow trout measured via ddPCR. ^c^ The rate of positive signals detected in quadruplicate for each sample. ^d^ The deviation of the mean content of rainbow trout measured via ddPCR compared to the actual value.

**Table 5:** Effect of food additives on the accuracy of ddPCR quantification of rainbow trout in Atlantic salmon.

Additives	Actual Value, %	Measured Value ^a^, %	RSD ^b^, %	Deviation ^c^, %
Sodium glutamate	70	68.23 ± 0.62	0.91	−2.52
β-carotene	70	71.34 ± 0.88	1.23	1.92
Carrageenan	70	69.91 ± 0.23	0.34	−2.99
Fish powder flavor	70	69.04 ± 0.58	0.83	−1.37

^a^ The content of rainbow trout measured via ddPCR. ^b^ The relative standard deviation of the content of rainbow trout measured via ddPCR. ^c^ The deviation of the mean content of rainbow trout measured via ddPCR compared to the actual value.

**Table 6:** Quantification results of commercially available Atlantic salmon products.

Sample	Processing Type	Declared Value of Rainbow Trout, %	Measured Value of Rainbow Trout, %	
			ddPCR	qPCR
Instant salmon_1	Steamed	0	0	0
Instant salmon_2	Steamed	0	0	0
Instant salmon_3	Steamed	0	0	0
Salmon sashimi_1	Smoked	0	0	0
Salmon sashimi_2	Smoked	0	0	0
Salmon sashimi_3	Smoked	0	0	0
Salmon sashimi_4	Raw	0	0	0
Salmon sashimi_5	Raw	0	100	100
Salmon fish ball_1	Frozen	0	0	0
Salmon fish ball_2	Frozen	0	62.50 ± 0.86	56.71 ± 2.00
Smoked salmon_1	Smoked	0	0	0
Smoked salmon_2	Smoked	0	0	0
Salmon fish sausage	Steamed	0	0	0
Grilled salmon	Grilled	0	0	0
Salmon fish floss	Steamed and dried	0	89.14 ± 1.74	82.84 ± 6.47
Canned salmon	Canned	0	0	0

## Data Availability

The data are contained within the article.
